# TNIP1‐mediated TNF‐α/NF‐κB signalling cascade sustains glioma cell proliferation

**DOI:** 10.1111/jcmm.14760

**Published:** 2019-11-05

**Authors:** Qingchun Lei, Huan Gu, Lei Li, Tingting Wu, Wentao Xie, Meizhang Li, Ninghui Zhao

**Affiliations:** ^1^ Neurosurgery Department The Second Affiliated Hospital of Kunming Medical University Kunming China; ^2^ Pu’er City People’s Hospital Pu’er China; ^3^ Biochemistry and Molecular Biology Laboratory School of Life Sciences Yunnan University Kunming China

**Keywords:** cellular signalling, Gliomas, NF‐κB, TNF‐α, TNIP1

## Abstract

As a malignant tumour of the central nervous system, glioma exhibits high incidence and poor prognosis. Although TNIP1 and the TNF‐α/NF‐κB axis play key roles in immune diseases and inflammatory responses, their relationship and role in glioma remain unknown. Here, we revealed high levels of TNIP1 and TNF‐α/NF‐κB in glioma tissue. Glioma cell proliferation was activated with TNF‐α treatment and showed extreme sensitivity to the TNF receptor antagonist. Furthermore, loss of TNIP1 disbanded the A20 complex responsible for IκB degradation and NF‐κB nucleus translocation, and consequently erased TNFα‐induced glioma cell proliferation. Thus, our investigation uncovered a vital function of the TNIP1‐mediated TNF‐α/NF‐κB axis in glioma cell proliferation and provides novel insight into glioma pathology and diagnosis.

## 1 INTRODUCTION

As a typical malignant tumour of the central nervous system, glioma affects 6.6 per million people and is characterized by very poor prognosis.^1^, ^2^ Glioma originates from neural stem cells, astrocytes or oligodendroglial progenitor cells.^3^ Though conventional hypothesis suggests the brain is an ‘immune privilege’ tissue, increasing studies indicate that brain disorders are affected by multiple immune factors, such as TNF‐α and NF‐κB.^4^, ^5^, ^6^ While ‘immune privilege’ is the main contributor to its poor prognosis, few effective therapeutic strategies exist for glioma due to the blood‐brain barrier.^7^ Glioblastoma patients have a poor survival of 14‐17 months in clinical investigations.^8^, ^9^, ^10^ Therefore, a broad understanding of the immune effector networks under a brain tumour context may provide new strategies for glioma therapy.

TNIP1, also known as ABIN1, is widely expressed in human tissues.^11^ TNIP1^−/−^ mice display normal Mendelian ratios before embryonic day 18.5 but die during late embryogenesis (2.4% born versus 25% expected) from foetal liver apoptosis, anaemia and hypoplasia.^12^ Furthermore, embryonic fibroblasts from TNIP‐1^−/−^ mice are hypersensitive to tumour necrosis factor (TNF)‐induced programmed cell death, with lethality reduced by crossing with mice without TNF receptor type I.^12^ Therefore, TNIP1 is essential for TNF‐induced programmed cell death in normal tissue.

Under a normal physiological context, TNF simultaneously induces pro‐apoptotic cascade triggered by caspase 8 cleavage and the anti‐apoptotic pathway mediated by nuclear factor kappa B (NF‐κB) activation.^13^ This TNF‐induced signalling contributes to cell survival or death, depending on signalling homoeostasis and cellular context.^14^, ^15^ In addition, cell survival and cell cycle are widely regulated by NF‐κB‐targeted genes.^16^, ^17^ IKK, which is comprised of IKK‐α, IKK‐β and IKK‐γ (also known as NEMO), induces phosphorylation and degradation of NF‐κB inhibitor‐α (IκB‐α). As a result, NF‐κB is liberated from suppressive conditions and is translocated into cellular nuclei for transcription of targeted genes.^18^, ^19^ Furthermore, TNIP1 also interacts with A20 (also known as TNFAIP3) and mediates the interaction of A20 with polyubiquitinated IKK‐γ.^20^ Previous studies have demonstrated that NF‐κB activity is inhibited by TNIP1 complexed with A20 and IKK through polyubiquitin binding, removing this polyubiquitin binding release NF‐κB and activating NF‐κB‐mediated signalling in pro‐inflammatory signalling pathways.^21^, ^22^

TNIP1 and TNFα‐induced NF‐κB activity play key roles in immune diseases and inflammatory responses.^23^ Genetic polymorphisms of TNIP1 are also reportedly correlated with elevated risk of gastric carcinoma or glioma prognosis 24, 25; however, the detailed roles of TNIP1 in glioma still remain to be elucidated. Here, we report that high levels of TNIP1 show a positive correlation with glioma cell proliferation and poor survival in glioma patients. Furthermore, RNA interference with TNIP1 expression in glioma cells markedly impacted proliferation activity. TNF‐α treatment elevated proliferation activity through NF‐κB signalling in normal glioma cells, but not in TNIP1‐down‐regulated glioma cells. These observations suggest that TNIP1 is essential for TNF‐α–induced cell proliferation in glioma progression and shows potential for targeted therapy in glioma patients.

## 2 RESULTS

### 2.1 High levels of TNIP1 correspond to poor survival in glioma patients

**Figure 1:**
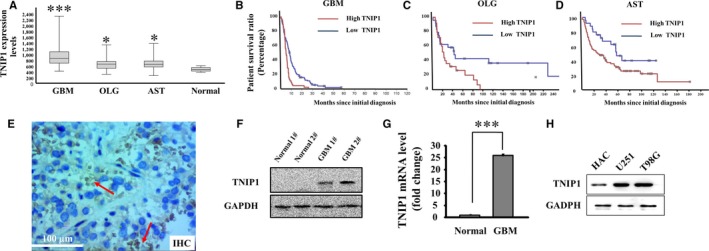
TNIP1 expression in various types of glioma. A, TNIP1 mRNA expression in GBM (214 samples, *P* = .0000284), OLG (66 samples, *P* = .0341), AST (145 samples, *P* = .0343) and normal brain (21) tissue samples (from the Rembrandt database). Different survival ratios of 178 GBM (B), 43 OLG (C) and 101 AST patients (D) with high or low TNIP1 expression (from the Rembrandt database). GBM, glioblastoma; OLG, oligodendroglioma; AST, astrocytoma. E, immunohistochemical examination of TNIP1 in glioblastoma. Western blot (F) and real‐time quantitative PCR (G separately confirmed protein and mRNA levels of TNIP1 in normal brain and glioblastoma tissues). Protein levels of TNIP1 in normal astrocyte (HAC), glioma (U251) and glioblastoma (T98G) cells (H)

To identify the correlation between TNIP1 expression and glioma progression, we determined the mRNA level of TNIP1 in glioma specimens. The Rembrandt data showed considerable increases in TNIP1 mRNA levels in different types of glioma tissue, including glioblastoma, oligodendroglioma and astrocytoma, compared with normal brain tissue (Figure 1A). High TNIP1 levels in glioma tissue were also observed in the Lee brain and Sun brain data (Figure S1A,B). Interestingly, glioma patients with high TNIP1 levels in all neoplasm tissues, including glioblastoma, oligodendroglioma and astrocytoma, showed significantly shorter survival compared with glioma patients with low TNIP1 levels (Figure 1B,1,1). Glioblastoma patients with the highest TNIP1 levels among the three types of glioma exhibited the poorest survival (less than 60 months) in both the high and low TNIP1 level groups (Figure 1A,1). To further verify the high TNIP1 levels in glioma tissue, we collected 20 glioblastoma samples and examined the expression of TNIP1 by immunohistochemical, immunoblot and quantitative polymerase chain reaction (qPCR) analyses (Figure 1E,F,G). Compared to the pericarcinomatous tissue, enhanced expression of TNIP1 was detected in glioblastoma tissue. Similarly, TNIP1 was also up‐regulated in two cancerous glial cells (Figure 1H). Furthermore, we imaged glioma tissue with the assistance of T1, T2 and contrast agent‐enhanced magnetic resonance imaging (Figure S2), with haematoxylin‐eosin (H&E) staining applied to verify carcinomatous brain tissue (Figure S3).

### 2.2 TNIP1 sustains glioma cell proliferation in vitro

**Figure 2:**
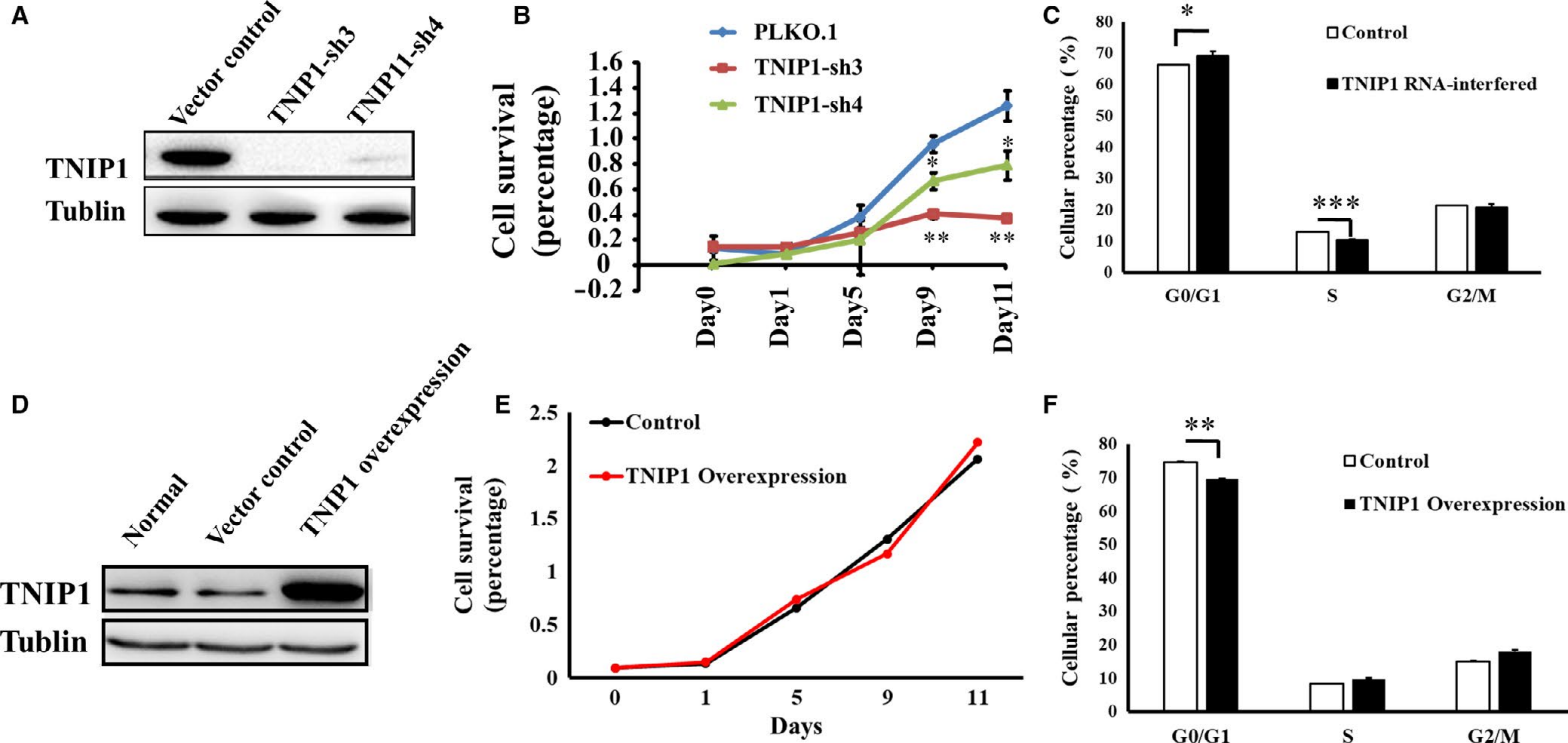
Role of TNIP1 expression in glioma cellular proliferation and cell cycle. Western blot analysis confirmed RNA interference efficiency (A) and overexpression (D) of TNIP1 in U251 glioma cells. Cellular proliferation activity in TNIP1‐RNA‐interfered (B) or TNIP1‐overexpressed (E) U251 cells. Cell cycle analysis in TNIP1‐RNA‐interfered (C) or TNIP1‐overexpressed (F) U251 cells

To evaluate the role of TNIP1 in glioma cell proliferation, we established stable TNIP1‐interfered U251 glioma cells. Immunoblot and qPCR analysis confirmed TNIP1 expression was significantly interfered (Figure 2A, Figure S4A). From day 5 after seeding, TNIP1‐interfered glioma cell lines (TNIP1‐sh3 and TNIP1‐sh4) showed distinct proliferation curves. Down‐regulation of TNIP1 dramatically retarded glioma cell proliferation (TNIP1‐sh3: *P* < .01; TNIP1‐sh4: *P* < .05) compared with the control cells (Figure 2B). Flow cytometry further demonstrated that TNIP1‐interfered glioma cells (TNIP1‐sh3) were arrested at the G0/G1 phase (*P* < .001) and resisted entry into the G2/M phase (*P* < .001) compared with the vector control (Figure 2C).

We also established stable TNIP1‐overexpressed U251 glioma cells. Immunoblot and qPCR analysis (Figure 2D, Figure S4B) showed no obvious change in U251 cell survival (Figure 2F). TNIP1‐overexpressed glioma cells were not arrested at the G0/G1 phase and were able to initiate cell cycle, with glioma cells at the S and G2/M phases not significantly increased (Figure 2F), illustrating that why TNIP1‐overexpressed glioma cells did not show increased cellular proliferation (Figure 2E).

### 2.3 Down‐regulation of TNIP1 improves survival of nude mice burdened with glioma

**Figure 3:**
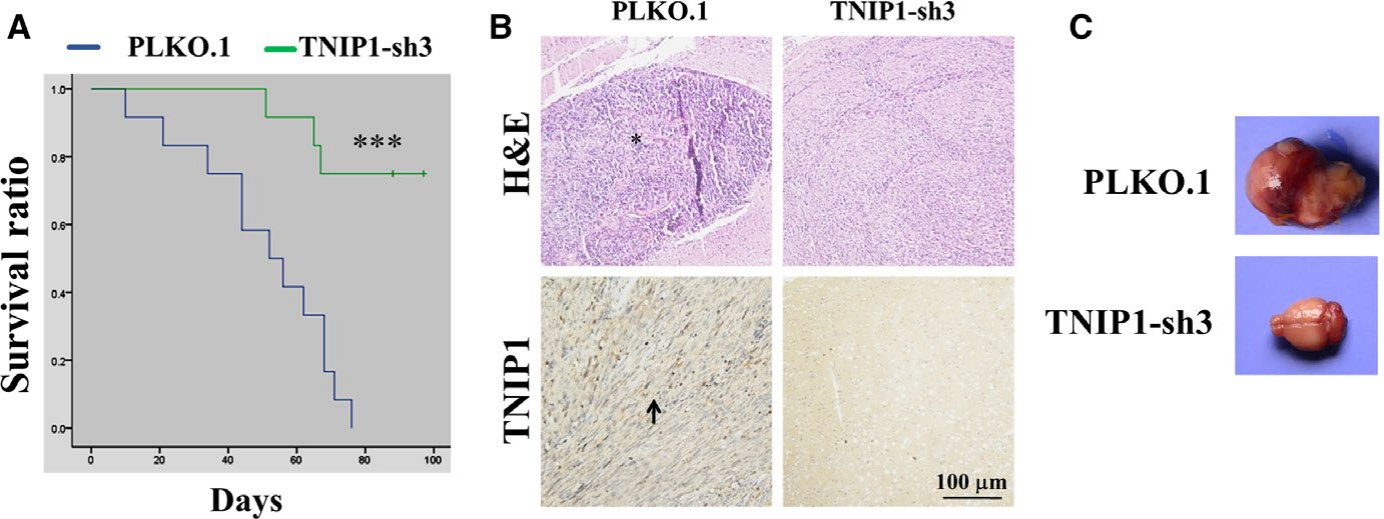
RNA interference with TNIP1 improved survival of nude mice burdened with glioma cell xenograft. A, survival curve of nude mice burdened with control or TNIP1‐down‐regulated U251 cells. Chi‐square test confirmed significant differences existed between the control and TNIP1‐down‐regulated group (*P* < .001). B, haematoxylin‐eosin (H&E) staining of mouse brain burdened with control or TNIP1‐down‐regulated U251 cells (up line). Immunohistochemical examination of TNIP1 in brain tissues burdened with control or TNIP1‐down‐regulated U251 cells (low line). C, Nude mouse brain xenografted with control (up panel) or TNIP1‐RNA–interfered (low panel) U251 glioma cells

We applied an orthotopic glioma xenograft strategy to explore the effect of TNIP1 down‐regulation on glioma tumour initiation and progression. All mice burdened with control U251 cells died before day 80. Within the same time range, only three mice (30%) died in the TNIP1‐sh3 group (*P* < .001) (Figure 3A). All nude mice were killed to collect brain tissue at day 100. H&E staining and immunohistochemical analysis confirmed the presence of neoplastic cells and TNIP1 expression in glioma xenografts of vector control cells (Figure 3B, left pane). Interestingly, no formative glioma tissue or TNIP1 expression was found in the TNIP1‐RNA interference group (Figure 3B right pane, C).

### 2.4 TNIP1‐regulated TNF‐α signalling participates in glioma cell proliferation

**Figure 4:**
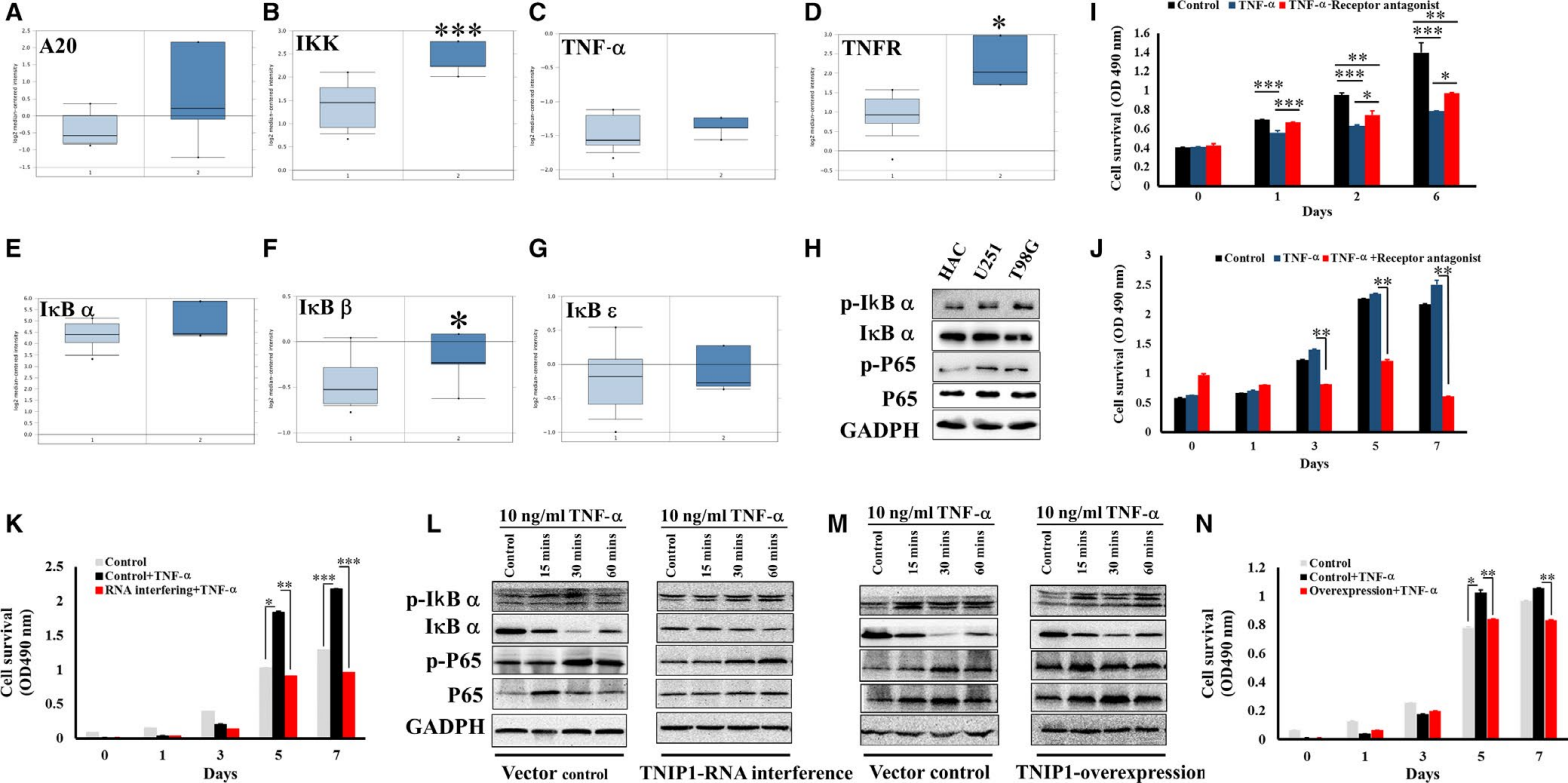
TNIP1 regulated the TNF‐α/NF‐κB signal axis in glioma cells. mRNA levels of TNIP1‐binding proteins, A20 (A) and IKK (B) in normal brain and glioblastoma tissues; mRNA levels of TNF‐α (C) and TNF receptor (D) in normal brain and glioblastoma tissues; mRNA levels of IκB subunit IκB‐α (E), IκB‐β (F), and IκB‐ (G) in normal brain and glioblastoma tissues (from TCGA database). 1, normal brain tissue; 2, glioblastoma tissue. A20: fold change = 1.754, *P* = .109; IKK: fold change = 2.023; *P* = 2.43E‐4; TNF‐α: fold change = 1.104, *P* = .072; TNFR: fold change = 2.466, *P* = .001; IκB‐α: fold change = 1.45, *P* = .089; IκB‐β: fold change = 1.208, *P* = .045; IκB‐: fold change = 1.151, *P* = .170. H, Phosphorylation of IkB α and P65 in normal astrocyte (HAC), glioma (U251) and glioblastoma (T98G) cells. Treatment with TNF‐α or TNF‐α receptor antagonist changed proliferation of HAC (**I)** and U251 (**J**) cells. K, Down‐regulation of TNIP1 quenched TNF‐α–induced cellular proliferation of U251. Down‐regulation of TNIP1 erased TNF‐α–induced degradation of IκB‐α and up‐regulation of P65 (L), but overexpression of TNIP1 did not alter protein levels of either IκB‐α or P65 (M). N, Overexpression of TNIP1 reduced TNF‐α–induced cellular proliferation of U251 cells

Because TNIP1 is involved in NF‐κB signalling in multiple immune diseases, we investigated several TNF‐α/NF‐κB signalling members in glioblastomas. As expected, TNIP1 complex components A20 and IKK were highly expressed in glioblastoma tissue (Figure 4A,4). In addition, TNIP1 signalling cascade components, including TNF‐α (Figure 4C), TNF receptor (Figure 4D) and three IκB subunits (Figure 4E,4,G), were significantly up‐regulated in glioblastoma tissue compared with normal brain tissue. We also investigated activation of IκB‐α and P65, a subunit of NF‐κB and phosphorylations of both proteins were increased in cancerous glial cells compared with normal astrocyte cells (Figure 4H). The integral activation of TNF‐α/NF‐κB suggested that it may be involved in glioblastoma progression. Therefore, we treated normal astrocyte cells with synthesized TNF‐α and found that cellular proliferation significantly decreased even TNF‐α receptor antagonist saved part of them (Figure 4I). Interestingly, same treatment to glioma cells induced converse phenotype. TNF‐α treatment promoted U251 cellular proliferation, and these cells were highly sensitive to the TNF‐α receptor antagonist (Figure 4J). Similar results were also observed in the T98G glioma cells (Figure S6A).

In our investigations, RNA interference with TNIP1 dramatically attenuated phosphorylation of NF‐κB subunit P65, but not IκB‐α. However, TNIP1 overexpression did not significantly increase P65 and IκB‐α phosphorylation in glioma cells (Figure S5). Further immunoblotting revealed that TNF‐α promoted the phosphorylation and degradation of IκB‐α in glioma cells in a time‐dependent manner, with NF‐κB subunit P65 increasingly expressed and phosphorylated in the same manner (Figure 4L, left pane). This indicates that TNF‐α may induce IκB degradation and consequently release NF‐κB from inhibitive status, resulting in the nuclear translocation of NF‐κB and rapid cell cycle and proliferation in glioma cells. In consideration of this possibility, we treated TNIP1‐interfered glioma cells with the same concentration of TNF‐α and same time course. In these cells, both IκB‐α degradation and P65 phosphorylation were eliminated (Figure 4L, right pane, Figure S6B) and TNF‐α–induced cell proliferation was erased (Figure 4K). However, excessive TNIP1 did not obviously alter TNF‐α–induced IκB‐α degradation and P65 phosphorylation (Figure 4M, Figure S6C). Surprisingly, excessive TNIP1 decreased TNF‐α–induced cell proliferation, though not as strikingly as TNIP1 down‐regulation (Figure 4N). Although the mechanisms remain to be further explored, the above results confirmed the key role of TNIP1 in TNF‐α–induced glioma cell proliferation.

**Figure 5:**
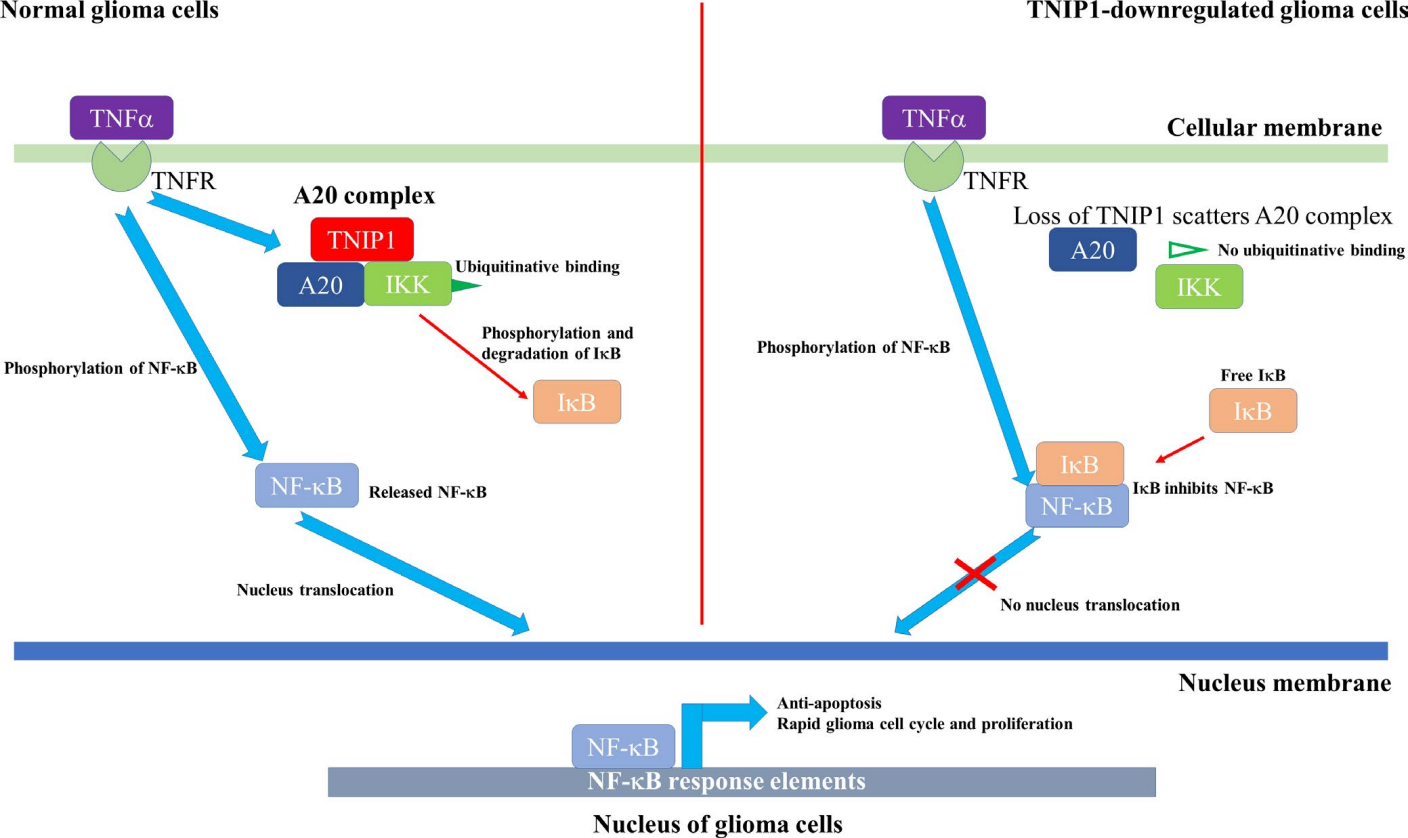
Proposed molecular mechanism of TNIP1‐regulated cell survival and proliferation of glioma cells. In normal glioma cells, NF‐κB cascade activator TNF‐α phosphorylates IκB‐α and induces degradation of IκB and thus liberates the NF‐κB signalling complex from IκB‐α inhibition, thereby activating glioma cell proliferation (left portion). In TNIP1‐down‐regulated glioma cells, loss of TNIP1 led to disbandment of the A20 complex and liberation of IκB from the complex. Free IκB prevented NF‐κB from translocating into the nucleus and thus subsequent cell proliferation (left portion)

In summary, high levels of TNIP1 in normal glioma cells supported the formation of the A20 complex, which freed IκB and led to inhibitive binding to NF‐κB. IκB‐binding disabled NF‐κB translocation into the glioma cell nucleus. In TNIP1‐down‐regulated glioma cells, loss of TNIP1 prevented the formation of the A20 complex and IKK was released from ubiquitin binding of the A20 complex. Liberated IKK phosphorylated and degraded IκB, with the free NF‐κB then phosphorylated by TNF‐α signalling and translocated into the glioma cell nuclei. As a result, NF‐κB activated its response elements responsible for glioma cell cycle arrest and proliferative inhibition (Figure 5). Thus, our investigation demonstrated that TNIP1 is an essential component for TNF‐α–induced phosphorylation and degradation of IκB‐α regulates glioma cell fate.

## 3 DISCUSSION

TNIP1 has been well studied in psoriasis, psoriatic arthritis, systemic lupus erythematosus (SLE) and systemic sclerosis^26^, ^27^, ^28^, ^29^, ^30^, ^31^, ^32^; however, it is poorly understood in cancer, especially in glioma. Our work revealed high levels of TNIP1 and its binding proteins, including A20 and IK, in glioma tissue, with its expression found to be negatively related to survival of glioma patients. Molecular and cellular study illustrated that down‐regulation of TNIP1 significantly damaged cell proliferation of glioma cells. TNIP‐interfered glioma cells exhibited G0/G1 phase arrest and restricted entry into the S phase. Therefore, this study provides direct experimental evidence of the relevance of TNIP1 in glioma progression.

Oshima et al reported that TNIP1 requires ubiquitin binding to restrict cell apoptosis.^33^ In addition, TNF has been implicated in cellular communication, cell differentiation and cell death, and is associated with diverse diseases, including cancers.^34^ Under normal physiological conditions, TNF signalling can elicit pro‐apoptotic or anti‐apoptotic pathways. For example, NF‐κB signalling is responsible for sustaining the anti‐apoptotic properties of Bcl_2_ and Bcl‐XL, the classical apoptosis carried out by caspase 9 and caspase 3.^35^, ^36^, ^37^, ^38^ In our observations, TNF‐α treatment immediately activated P65 (a subunit of NF‐κB) in normal glioma cells, which was remarkably attenuated in the TNIP1‐interfered glioma cells. These results indicate that TNIP1 is involved in glioma proliferation via TNF‐α/NF‐κB signalling.

Previous research has reported that NF‐κB signalling up‐regulates A20 expression, which, in turn, deubiquitinates NF‐κB, leading to inhibition of NF‐κB signalling as a negative feedback.^39^ Earlier studies have also illustrated that NF‐κB response elements are located at the promoter region of TNIP1 (44) and that overexpression and activation of NF‐κB elevates TNIP1 mRNA levels.^32^, ^40^, ^41^, ^42^, ^43^ Surprisingly, in our study, TNF‐α signalling did not increase the protein levels of TNIP1 or A20 (data not shown) but did induce degradation of IκB‐α. In addition, TNIP1‐RNA interference decreased the phosphorylation and degradation of IκB‐α in glioma cells. Therefore, we supposed that TNF‐α signalling induced the formation of a complex comprised of A20, TNIP1 and IKK, and this complex phosphorylated and induced IκB‐α degradation, leading to liberation of NF‐κB from the inhibitive condition in normal glioma cells. This suggests that TNIP1 is a key regulator in the TNF‐α signalling pathway in glioma tissue.

Ubiquitin binding and the NEMO (UBAN) domain of mutant TNIP1 fail to interact with linear deubiquitin chains, leading to liberation of the NF‐κB signalling pathway.^44^ The UBAN domain is reported to interact with both linear and Lys 63‐linked chains under various cellular contexts, but with a preference for linear deubiquitin chains.^45^, ^46^, ^47^, ^48^, ^49^ In our work, however, TNF‐α–induced NF‐κB activation benefited from high levels of TNIP1, and down‐regulation of TNIP1 reduced P65 activation in glioma cells. The glioma cell phenotype may originate from malignant transformation of brain tissue. In addition, whether TNIP1 regulates the NF‐κB signalling pathway via ubiquitin binding and which domain(s) are responsible for this regulation in glioma cells are unclear. Thus, additional research is required to interpret the related mechanisms.

Collectively, our study showed high levels of TNIP1 in glioma tissue, which were further correlated with poor survival in glioma patients. Down‐regulation of TNIP1 decreased glioma cell proliferation, in which the TNF‐α/NF‐κB signalling pathway was involved. These findings will benefit pathological diagnosis and TNIP1‐targeting therapeutics in glioma patients, even though much work remains to be carried out.

## 4 MATERIALS AND METHODS

### 4.1 Cell culture

Glioma cell lines U251 and T98G were cultured in Dulbecco's modified Eagle's medium (DMEM, Gibco, China) supplemented with 10% foetal bovine serum (FBS, Biological Industries, Australia) in an incubator with 5% CO_2_ at 37°C.

### 4.2 Flow cytometry

Glioma cells were harvested and incubated in a solution containing 50 μg/mL of propidium iodide (PI) for 30 minutes at 4°C under light‐free conditions. For each population, at least 1 × 10^4^ cells were analysed by fluorescence‐activated cell sorting (FACS) with a flow cytometer (Accuri C6, Becton‐Dickinson, USA). The proportion of cells in the G0/G1, S and G2/M phases was calculated using BD Accuri C6 software.

### 4.3 TNF‐α treatment

Lyophilized TNF‐α protein (Abcam, USA) was reconstituted with dH_2_O to a final concentration of 1.0 mg/mL and then added into culture medium for a working concentration of 10 ng/mL.

### 4.4 Western blot analysis

Rabbit anti‐human tubulin antibody was purchased from Beyotime Biotechnology (China). Rabbit anti‐human p65 and phospho‐p65 (Ser536) antibodies were obtained from Cell Signaling Technology (USA). Rabbit anti‐human IκB‐α and phospho‐IκB‐α (Ser36) and TNIP1 antibodies were purchased from Abcam (USA). Western blots were conducted according to standard procedures.

### 4.5 Immunohistochemistry

The clinical glioma specimens were obtained from the Neurosurgery Department, the Second Affiliated Hospital of Kunming Medical University, China. Immunohistochemical staining was conducted according to standard procedures. Briefly, paraffin‐embedded sections were incubated with antibodies of rabbit anti‐human TNIP1 (Abcam, USA) overnight at 4°C. The sections were then further stained with goat anti‐human secondary antibodies conjugated with horseradish peroxidase (Santa Cruz Biotechnology, USA). The immunohistochemical signals were developed with DAB reagent (Boster Biological Technology Ltd., China) and further examined under a light microscope (Olympus, Japan).

### 4.6 qPCR

Total RNA was purified from cells using RNAiso Reagent (TaKaRa, China), followed by reverse‐transcription (RT) reactions to obtain cDNA using a Prime Script TM RT Reagent Kit (TaKaRa, China). SYBR Primix Ex Taq TM (TaKaRa, China) was used for real‐time fluorescence PCR (qPCR). The following primers were used: TNIP1 forward primer sequence: 5′AGGTCACCCTGTCAAATGCC3′; reverse primer sequence: 5′GCTCCACATGGTAACGCTCT3′. 18S forward primer sequence: 5′CAGCCACCCGAGATTGAGCA3′; reverse primer sequence: 5′TAGTAGCGACGGGCGGTGTG3′. All experiments were carried out using real‐time PCR equipment (Applied Biosystems, USA).

### 4.7 Cell proliferation assay

Cells were seeded into 96‐well plates at a density of 500 cells per well. The next day, 10 ng/mL of active TNF‐α (Abcam, USA) was used to treat the cells for 15, 30 or 60 minutes. Cell proliferation was analysed by a Cell Titer 96® Aqueous One Solution Cell Proliferation Assay Kit (Promega, USA). After adding the reagent to the cell culture well and incubating for 2 hours, we determined cell proliferation based on colour changes, with a Spectra Max M2 (Molecular Devices, USA) used to test optical density (OD) values at 490 nm.

### 4.8 Knockdown of TNIP1 mRNA

TNIP1 shRNA/PLKO.1 and PLKO.1 empty vectors were purchased from Sigma (USA). To knock down the mRNA expression level of TNIP1, two stably interfered cell lines (sh3 and sh4) were established according to standard experimental procedures. Briefly, TNIP1 shRNA/PLKO.1 (or PLKO.1) plasmids together with pMD2.G and psPAX2 plasmids were mixed with Fu GENE HD transfection reagent (Roche, Germany) and incubated at room temperature for 20 minutes, with the mixture then applied for transfecting 293T cells. Lentiviral particles were harvested 48 hours after transfection and added to glioma cells for an additional 48 hours. Stable U251 cells were further screened with 10 µg/mL of puromycin (Tocris, UK) for 7 days.

### 4.9 Magnetic resonance imaging (MRI)

MRI examinations were performed on a three Tesla Magnetom Trio MRI system (Siemens Medical Solutions, Germany). T1‐weighted images: axial images were acquired before and after contrast agent injection (gadopentetate‐dimeglumine, Magnevist, Bayer Schering Pharma AG). Repetition time 600 ms; echo time 12 ms; slice thickness 5 mm; interslice distance 1 mm; in‐plane resolution 0.45:0.45 mm; matrix size 384:512; and 23 slices. T2‐weighted images: axial images with repetition time 10 seconds, echo time 70 ms, slice thickness 5 mm, interslice distance 1 mm, in‐plane resolution 0.60:0.45 mm, matrix size 384:512 and 23 slices. Dynamic contrast‐enhanced images: axial, fast gradient‐echo images with repetition time 5.7 ms, echo time 2.73 ms, slice thickness 2.1 mm, interslice distance 0.4 mm, in‐plane resolution 2.90:2.00 mm, matrix size 128:87 and 20 slices. After approximately 52 seconds of imaging, a 0.1 mmol/kg dose of Gd‐DTPA was injected at 5 cc/s. Spoiled gradient recalled‐echo images with five different flip angles (2°, 5°, 10°, 15° and 30°) were also initially acquired for T1 mapping.

### 4.10 Orthotopic xenograft of glioma cells

Immune‐deficient mice (Nu/Nu, male, 6 weeks old) were purchased from Beijing Vital River Laboratory Animal Technology Co., Ltd (China) and were maintained in a specific pathogen free (SPF) room. To establish orthotopic glioma, 100,000 U251 cells were stereotactically injected in a 1 mL volume into the left striatum over 1 minute into the following coordinates: 1 mm anterior, 1 mm lateral from bregma and 3 mm deep from cortical surface. Each group contained 10 mice for the survival experiments. Dates of mouse deaths were recorded for the survival curve analysis.

### 4.11 Bioinformatics

The Oncomine (http://www.oncomine.org) and Rembrandt databases (http://www.rembrandt.nci.nih.gov) were used for clinical sample analysis. Relevant analytical parameters are provided in the figures.

### 4.12 Ethics committee approval

The Ethics Committee of the Second Affiliated Hospital of Kunming Medical University approved this study. Under this supervision, written informed consent from the donors or next of kin was obtained for the use of samples in this research.

### 4.13 Statistical analysis

All experiments were repeated at least three times. Statistical analyses were performed using SPSS 13.0 software. Cell proliferation and qPCR were assessed by one‐way analysis of variance (ANOVA).

## COMPETING INTERESTS

The authors declare that they have no competing interests.

## AUTHORS’ CONTRIBUTIONS

ZN and LM designed and supervised the study, handled the sample, analysed the data and wrote the manuscript; LQ and GH: collected the samples and carried out the experiments. LL, WT and XW: collected the samples and patient information.

## ETHICS APPROVAL AND CONSENT TO PARTICIPATE

The Ethics Committee of the Second Affiliated Hospital of Kunming Medical University approved this study. Written informed consent from all donors or next of kin was obtained prior to the use of patient samples in this research.

## CONSENT FOR PUBLICATION

Not applicable.

## AVAILABILITY OF DATA MATERIALS

All data generated or analysed during this study are included in this article and its additional files.
