# Supplementary figures and images for: TNIP1‐mediated TNF‐α/NF‐κB signalling cascade sustains glioma cell proliferation
Source: J Cell Mol Med. 2019 Nov 5;24(1):530–8. doi: 10.1111/jcmm.14760 (PMC6933386; doi:10.1111/jcmm.14760)

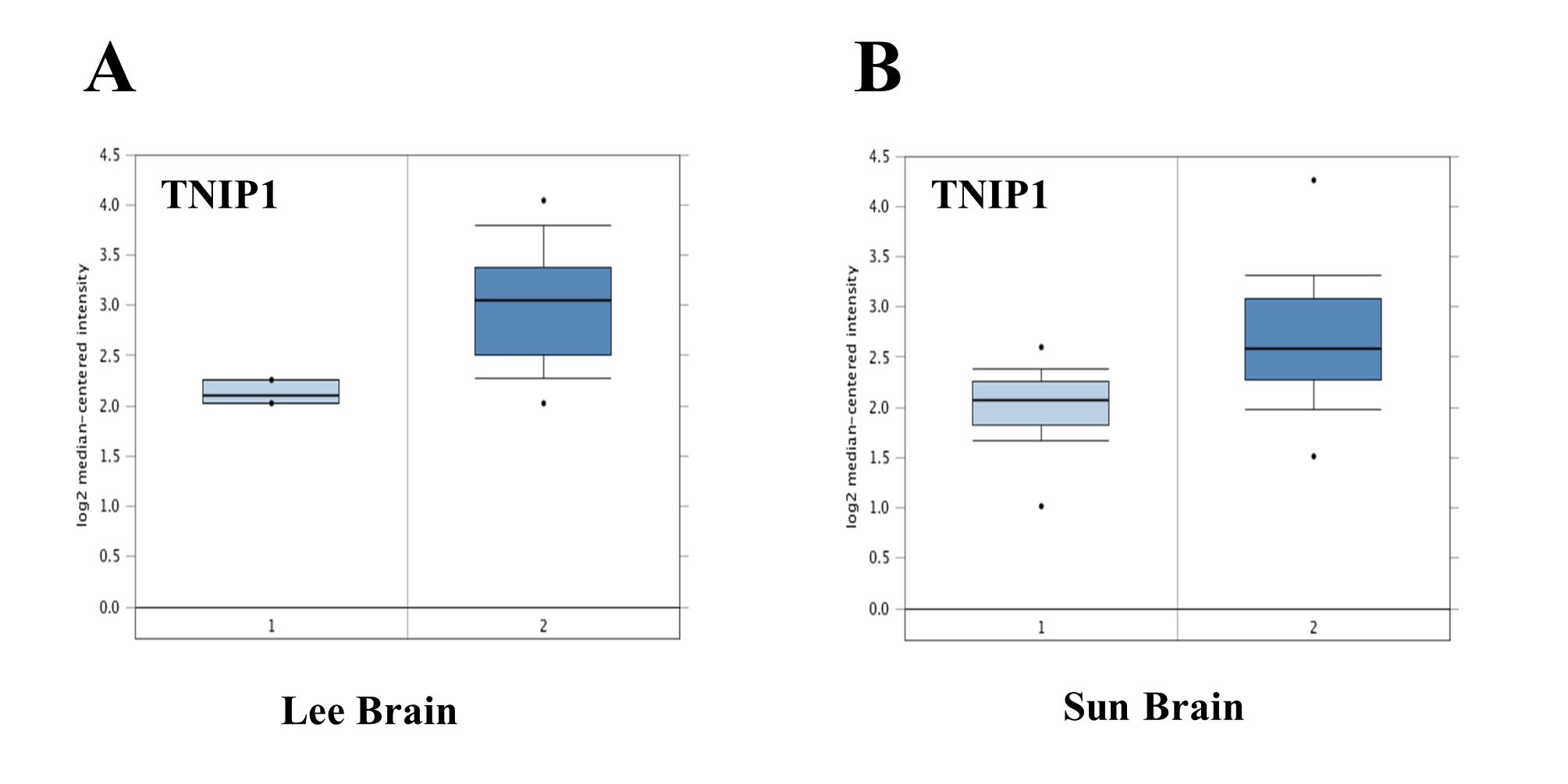

Supplement: Supplementary file 1 [file JCMM-24-530-s001.TIF]

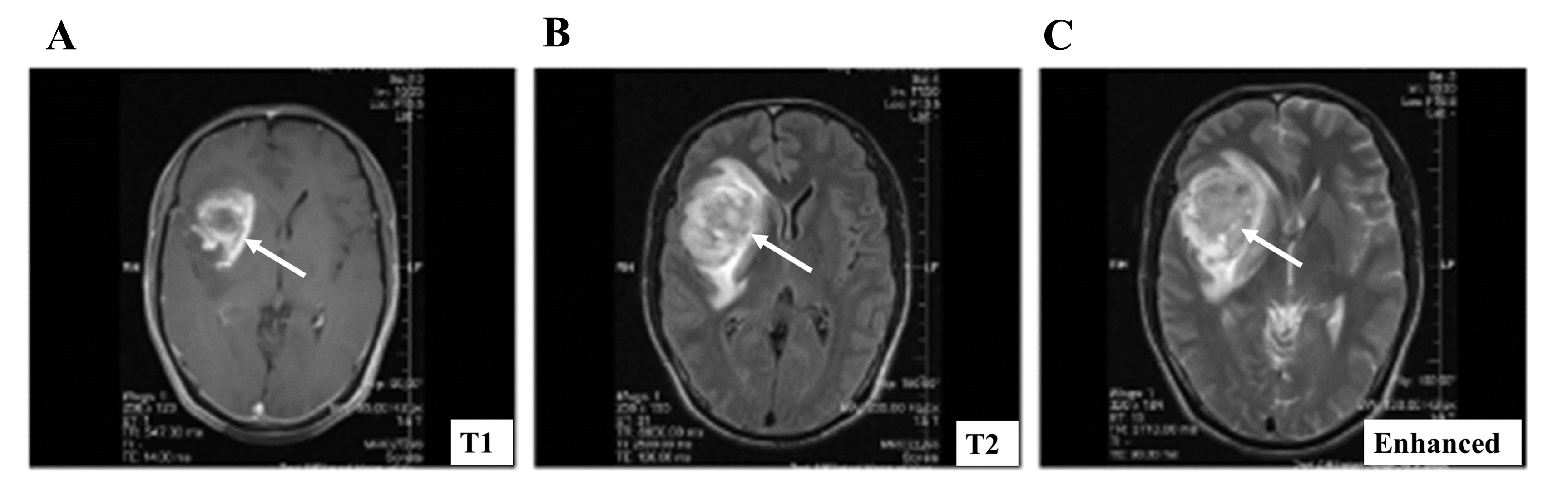

Supplement: Supplementary file 2 [file JCMM-24-530-s002.TIF]

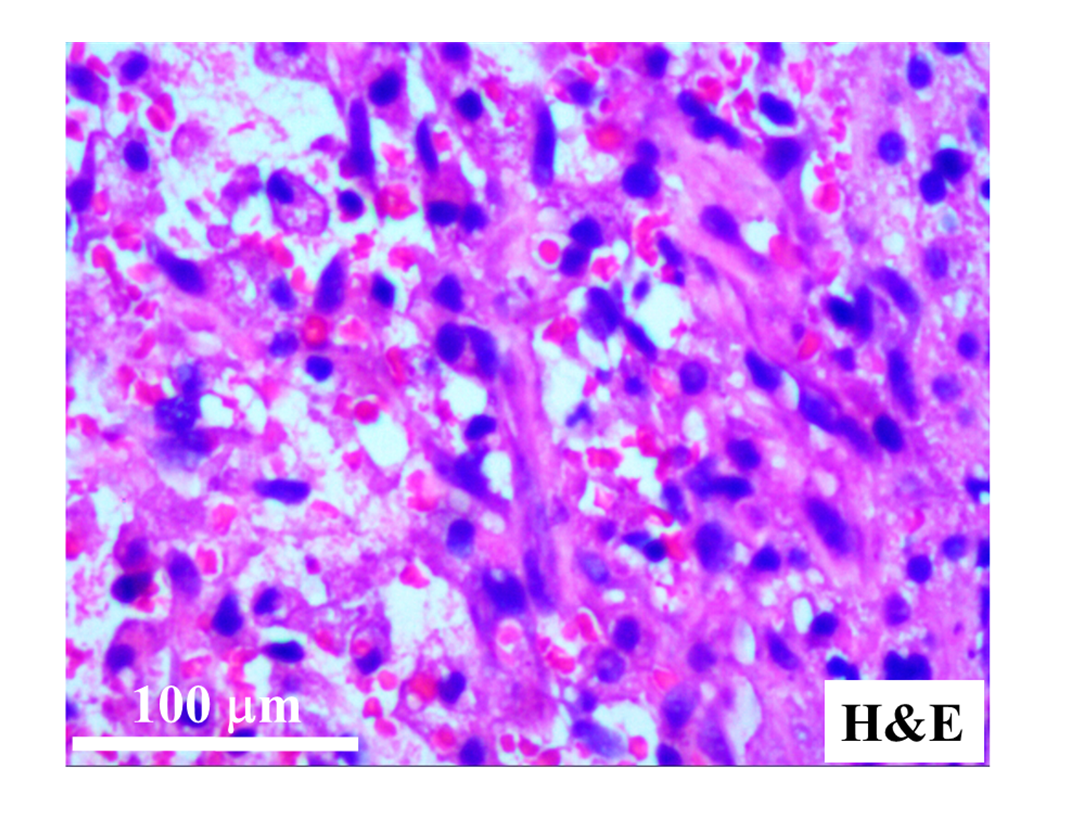

Supplement: Supplementary file 3 [file JCMM-24-530-s003.TIF]

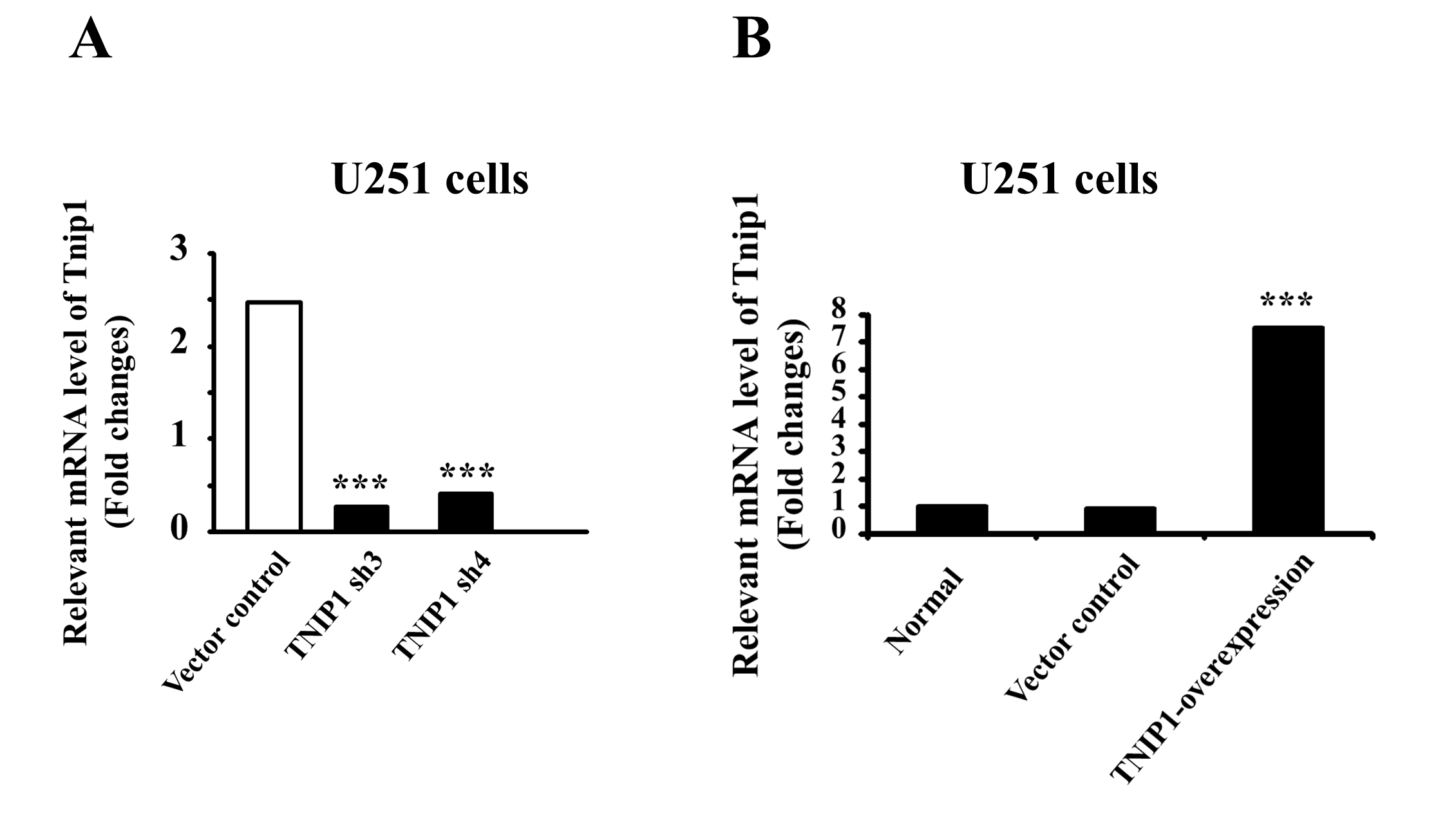

Supplement: Supplementary file 4 [file JCMM-24-530-s004.TIF]

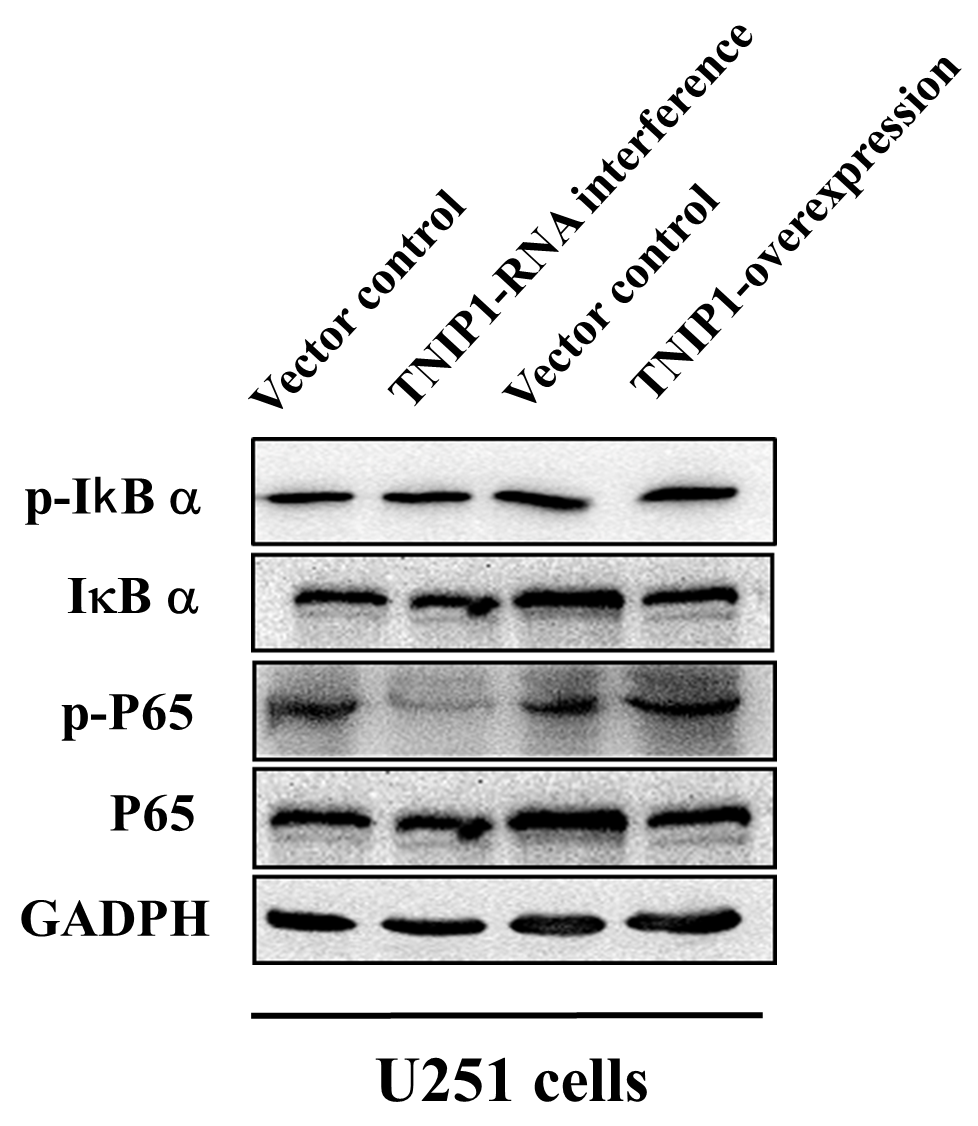

Supplement: Supplementary file 5 [file JCMM-24-530-s005.TIF]

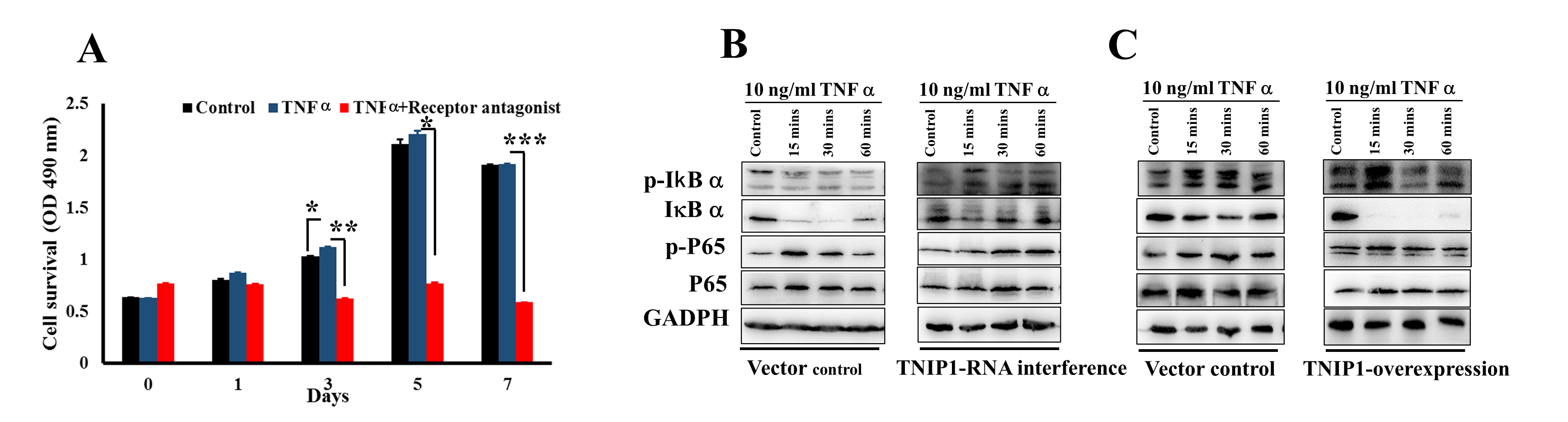

Supplement: Supplementary file 6 [file JCMM-24-530-s006.TIF]
