# Supplementary material for: TNIP1‐mediated TNF‐α/NF‐κB signalling cascade sustains glioma cell proliferation
Source: J Cell Mol Med. 2019 Nov 5;24(1):530–8. doi: 10.1111/jcmm.14760 (PMC6933386; doi:10.1111/jcmm.14760)
Supplement: Supplementary file 7 [file JCMM-24-530-s007.docx]

**Supplementary Materials**

**Figure S1**. mRNA levels of TNIP1 in normal brain and glioblastoma (GBM) tissues from Lee Brain (A) and Sun Brain (B) databases. Lee Brain data: fold change = 1.788; *P* = 6.18E-6; normal brain (3); glioblastoma (22). Sun Brain data: fold change = 1.553; *P* = 4.53E-9; normal brain (23); glioblastoma (81). 1, normal brain tissue; 2, glioblastoma tissue.

**Figure S2**. Magnetic resonance imaging (MRI) of glioblastoma (GBM) tissue by T1 (A), T2 (B), and enhanced (C) technologies.

**Figure S3**. Hematoxylin & eosin (H&E) staining of glioblastoma (GBM) specimens.

**Figure S4**. mRNA levels in TNIP1-RNA-interfered (A) and TNIP1-overexpressed (B) glioma cells of U251 determined using real-time quantitative PCR.

**Figure S5**. Phosphorylation of P65 and IκB α in TNIP1-RNA-interfered or TNIP1-overexpressed glioma cells of U251.

**Figure S6**. A, Treatment with TNFα or TNF receptor antagonist changed T98G cell proliferation. TNFα-induced cellular signaling cascade in TNIP1-RNA-interfered (B), or TNIP1-overexpressed (C) glioma cells of T98G.
